# Structural and functional insights into ABHD5, a ligand-regulated lipase co-activator

**DOI:** 10.1038/s41598-021-04179-7

**Published:** 2022-02-16

**Authors:** Yan Yuan Tseng, Matthew A. Sanders, Huamei Zhang, Li Zhou, Chia-Yi Chou, James G. Granneman

**Affiliations:** 1 Center for Molecular Medicine and Genetics, Wayne State University School of Medicine, Detroit, MI 48201 USA; 2 Karmanos Cancer Institute, Wayne State University School of Medicine, 4100 John R, Detroit, MI 48201 USA; 3 Center for Integrative Metabolic and Endocrine Research, Wayne State University School of Medicine, Detroit, MI 48201 USA

**Keywords:** Metabolomics, Fats, Molecular modelling, Single-molecule biophysics

## Abstract

Alpha/beta hydrolase domain-containing protein 5 (ABHD5) is a highly conserved protein that regulates various lipid metabolic pathways via interactions with members of the perilipin (PLIN) and Patatin-like phospholipase domain-containing protein (PNPLA) protein families. Loss of function mutations in ABHD5 result in Chanarin–Dorfman Syndrome (CDS), characterized by ectopic lipid accumulation in numerous cell types and severe ichthyosis. Recent data demonstrates that ABHD5 is the target of synthetic and endogenous ligands that might be therapeutic beneficial for treating metabolic diseases and cancers. However, the structural basis of ABHD5 functional activities, such as protein–protein interactions and ligand binding is presently unknown. To address this gap, we constructed theoretical structural models of ABHD5 by comparative modeling and topological shape analysis to assess the spatial patterns of ABHD5 conformations computed in protein dynamics. We identified functionally important residues on ABHD5 surface for lipolysis activation by PNPLA2, lipid droplet targeting and PLIN-binding. We validated the computational model by examining the effects of mutating key residues in ABHD5 on an array of functional assays. Our integrated computational and experimental findings provide new insights into the structural basis of the diverse functions of ABHD5 as well as pathological mutations that result in CDS.

## Introduction

ABHD5 (αβ hydrolase domain-containing protein 5) is a highly conserved protein that plays diverse roles in lipid metabolism including lipolysis in adipose tissue and muscle^1,2^, lipid barrier formation in skin^3,4^, Hepatitis C Virus assembly and production^5^, and suppression of tumor progression in colon and prostate cancer^6,7^. However, the mechanisms by which ABHD5 influences such diverse processes are not well understood, but likely involve interactions with specific binding partners and lipid ligands.

Although ABHD5 is a member of the αβ hydrolase family, the conserved serine nucleophile of closely-related paralogs has been substituted by asparagine; thus, ABHD5 has no known independent hydrolase activity. Presently, the best known function of ABHD5 is that of a coactivator of Patatin-like phospholipase domain-containing protein 2 (PNPLA2, aka Adipose Triglyceride Lipase, ATGL), the rate-limiting triglyceride lipase enzyme in adipose tissue, liver and heart. Because loss of ABHD5 function does not precisely phenocopy loss of PNPLA2, ABHD5 likely regulates additional pathways. Indeed, very recent work indicates that ABHD5 alters the functional activities of other PNPLA family members, including PNPLA1^4^ and PNPLA3^8^. Furthermore, ABHD5 interacts with certain members of the perilipin (PLIN) family of lipid droplet scaffold proteins to regulate subcellular targeting and interactions with effector proteins, like PNPLA2^9–11^. In this regard we recently demonstrated that binding of long chain acyl-Coenzyme A^12^ to ABHD5 promotes its interactions with PLIN proteins and suppresses interactions with PNPLA2. Thus, it seems likely that duplication of the ancestral gene allowed evolution of novel functional domains/surfaces of ABHD5 that sense metabolic status, regulate protein–protein interactions, and control substrate accessibility of PNPLA family members, yet do not require serine hydrolase activity.

To better understand a structure–function relationship of ABHD5, we sought to computationally and experimentally identify and characterize its binding surfaces that are linked to its biological functions. We applied homology modeling to ABHD5 primary sequence to construct a structural model. We took the advantages of molecular dynamics (MD) simulations^13^ to refine the initial model and further explored a variety of conformations that might not be evident in native models or structures. After MD refinement, we utilized Alpha-Shape theory^14–19^ to conduct shape analysis and perform surface perturbation on our ABHD5 structural models. To validate our predicted model, we assessed the effects of site-directed mutagenesis on the core functions of ABHD5, including LD targeting, PLIN binding and PNPLA2/ATGL activation. Lastly, we mapped the geometric locations of disease-causing mutations of human ABHD5 that result in Chanarin–Dorfman Syndrome (CDS)^20–22^, a genetic defect of neutral lipid metabolism^3^. Together, our structure model and experimental tests provide important new clues to facilitate the understanding of ABHD5 molecular functions in metabolic control, skin barrier formation, Hepatitis C Virus morphogenesis and tumor progression.

## Results

### Computational structural model of ABHD5

#### Shape analysis of a structural model

**Figure 1:**
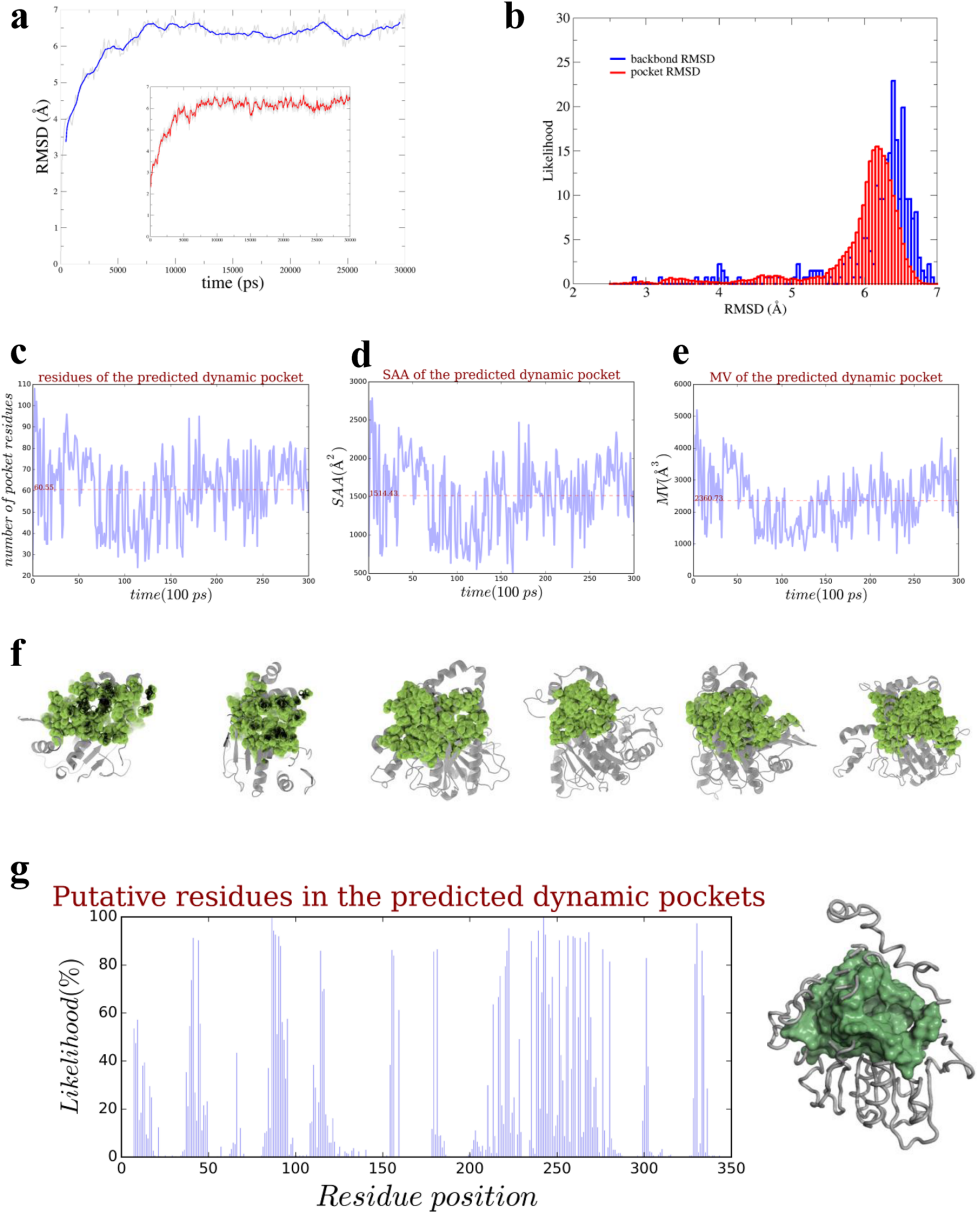
Assessing the dynamic surface of ABHD5 in MD simulations. (**a**) During MD simulations of 30 ns, ABHD5 dynamic conformations in a trajectory were sampled and refined. The backbone (global) and predicted main binding pocket (local) of RMSD values range within 7 Å. Their RMSD distributions of backbone (blue) and binding-pocket (red) were computed in (**b**) with a mean of 6.2 Å and 5.9 Å, respectively. The trivial difference of RMSD indicates that the binding pocket reshapes along with the conformation of the backbone. In each 100 ps step, the geometric measurement of the dynamic pocket was assessed, including the mean number (61 amino acid residues) of pocket residues in (**c**), its mean molecular volume (MV, 2360 Å^3^) in (**d**) and mean solvent accessible areas (SAA, 1514 Å^2^) in (**e**) during simulations. In structural analysis of structural alignment on the 300 conformers, classification was performed based on the customized cutoffs with incremental decreases from 3.5 to 1.5 Å of RMSD. Six subclasses were classified for shape analysis. For each of subclasses, a representative in (**f**) with the middle RMSD was selected with its identified binding pocket (green). Each residue of ABHD5 was assigned with a probability of appearing as a pocket residue on the dynamic surfaces (green) of the 300 conformers. (**g**) How a pocket residue emerged from a residue on the surface or vice versa is recorded in the dynamic conformers. By identifying the geometric locations of a residue in all 300 conformers, the likelihood of a pocket residue is computed for dynamically occupying a pocket in the trajectory.

To gain insights into structure–function relationships of ABHD5, we built an initial computational structural model for mouse ABHD5 based on homology modeling^23^. To further refine the initial model, we sampled diverse conformations using MD simulations^13^ (Fig. 1a,b). For each structural conformer at a given time, we identified the structural feature of a pocket using the weighted 3D Alpha Shape Delaunay triangulation with the flow algorithm^14–16,19^. After partitioning the surface of ABHD5, we identified more than 30 putative pockets^14–16,24^. For each pocket, we extracted residues and determined the spatial pattern (a substring of pocket residues). We further computed the molecular volumes (MV) and solvent accessible areas (SAA) of a pocket. As shown in Fig. 1c–e, each of these conformers possesses a main binding pocket that contains a mean of 61 binding residues, a mean of molecular volume of 2360 Å^3^ and a corresponding solvent accessible area of 1514 Å^2^. Geometric calculations also indicated that the binding pocket has an elongated shape with a corresponding MV correlated with SAA. Following pairwise structural alignment of conformers at a cutoff RMSD (Root Mean Square Deviation) value of 0.25 Å, the diversity of dynamic pocket conformations could be divided into six subclasses. The detailed geometric measurement of each subclass allowed us to identify putative functional pockets on the ABHD5 surface and further extract functionally important residues. As shown in Fig. 1d, the simulated MVs in ABHD5 binding pockets fluctuate over time. These dynamic shapes implied that the geometric locations of atoms will differ across these conformers during simulations. Using dynamic shape analysis^14–16^, we then computed geometric locations of pockets, surfaces, and cores of these six clustered conformers in a trajectory. The binding surface structural features of representative conformers are presented in Fig. 1f.

To further assess the spatial patterns of a dynamic binding pocket, we computed the probability of specific residues occupying a pocket (Fig. 1g). As an example, Y330 has 97.3% likelihood of occupying the binding pocket of conformers, whereas D334 has 67.3%, indicating Y330 constantly appears as a pocket residue on the ABHD5 surface. Although the crystal structures of ABHD5 are unknown, we took advantage of the surface perturbation during simulations to sample and reconstruct putative shapes of ABHD5 and characterize its functionally important residues that might be involved in biological activity.

#### Dissecting a structural model

**Figure 2:**
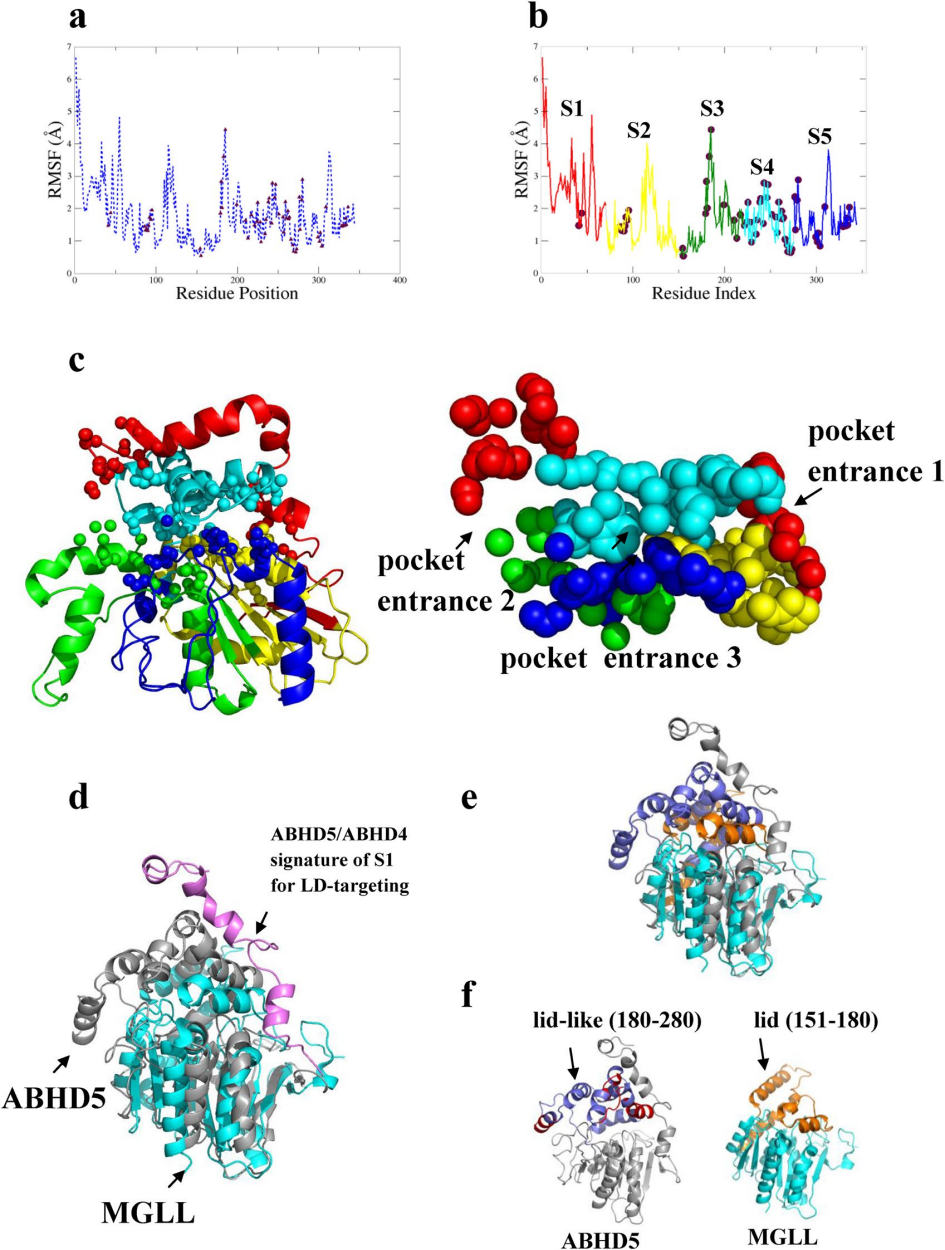
ABHD5 conformations with RMSF analysis. Structural comparisons of conformers were performed to assess residue dynamic movement and contact during MD simulations. (**a**) Pocket residues (brown) exhibited relatively constrained (< 2.5 Å) values of RMSF compared to those of the backbone (blue). Based on the RMSF, the model can be divided into five segments of S1, S2, S3, S4, and S5 as in (**b**). Subsequently, the surface areas of the identified pocket with structural features e.g., pocket mouths were also separated correspondingly in (**c**). The structural alignment between ABHD5 (grey) and MGLL (monoglyceride lipase, E.C 3.1.1.23) in cyan reveals that S1 is a characteristic structural feature (pink) in (**d**) of LD-targeting that has undergone the evolution of subcellular localization on LD membrane. Among ABHD family members, ABHD5 and ABHD4 only have capacity of LD translocation. The structural comparison further unveiled that the distinctive lid (orange) of MGLL is superimposed with the putative lid (blue) in (**e**) of ABHD5 which has a significantly larger lid-like structural feature in (**f**) with additional charged and hydrophobic residues that might be involved in regulation or protein–protein interaction.

Next we assessed all pairwise residue contact profiles using MD simulations. As shown simulations in Fig. 2a, we compared the structures of conformers at different time steps in a trajectory. Most of pocket residues (brown spheres) exhibited fluctuations of < a RMSD of 2.5 Å measured by root mean square fluctuation (RMSF) in the trajectory^13^. The RMSF measurement indicates that our predicted model is structurally compact and folded as Alpha Beta 3-Layer aba Sandwich^25^ (CATH: 3.40.50.1820) of its core structures that have been folded properly^23,26^. More importantly, the identified pockets significantly agree with structural evidence that functionally important residues tend to cluster closely to form a specific shape, such as a pocket or shallow depression area^18,19^. These structural features can be detected on a protein surface by our topology-based method. In contrast, conformational changes of RMSF of > 3 Å mostly came from rearrangements of multiple loops or unstructured regions. Based on the RMSF cutoff of 3 Å for separation, we further refined the model into 5 segments which also divided the binding pocket correspondingly shown spheres in Fig. 2b, where pocket residues appear as brown spheres. Note that at N terminus and C terminus are expected to have higher RMSF values as both termini tend to form flexible loops. The sequence starts from 1 to 70 (red, S1), then to 71 to 150 (yellow, S2), green (151 to 220, S3), 221 to 275 (cyan, S4) and ends in 276 to 350 (blue, S5) segments. The red segment (S1) is the most flexible and dynamic one with the highest RMSF at N terminus. The yellow and green (S2, S3) segments are composed of all beta sheets known to build the protein core and maintain its structural stability. As shown in Fig. 2c, the surface areas of the binding pocket appear mostly in the green, cyan and blue (S3, S4, S5) segments.

In our shape analysis by the flow algorithm^14–16^, the predicted binding pocket (Fig. 2c) contains three entrances: pocket entrance-1 and entrance-2 are symmetrically regulated by yellow, and green (S2, S3) segments, whereas the main entrance-3 is modulated by both cyan and blue (S4, S5) segments. These structural features suggest that ligands/substrates would potentially contact the entrance residues of the predicted binding pocket.

Compared with other ABHD family members (e.g., MGLL (monoglyceride lipase), ABHD6 and ABHD12), the S1 segment, which is composed of loops and short-helices (colored violet in Fig. 2d), is exclusive to close paralogs ABHD5 and ABHD4, suggesting a conserved role in subcellular targeting to intracellular lipid droplets (LD). Our structural alignment across species also reveals that the 1–50 residues of S1 segment possesses a unique feature only found in ABHD5 othologs. Removal of the first 40 residues in S1 prevents targeting of ABHD5 to intracellular lipid droplets^27^, which is thought to be mediated by a hydrophobic cluster of Trp residues (W21, W25, and W29).

The S2 segment is highly conserved in the αβ hydrolase family. The structural alignment of superimposition of ABHD5 and MGLL shows a small RMSD of 4.2 Å, indicating these proteins are structurally related. As shown in Fig. 2e, the S2 segment constructs the protein core with beta sheets connected by loops and helices. The catalytic center of nucleophilic Ser (e.g., ABHD4 or MGLL) or inactive Asn (ABHD5) is structurally conserved and situated on the edge of a helix from N^155^ to K^168^ of the S2 segment. Extending from the protein core, the S3 and S4 segments create an amphipathic lid-like domain, which is a characteristic structural feature of many lipases^28,29^. Comparison of the lid-like domains of ABHD5 and MGLL (Fig. 2f) revealed that the lid-like domain of ABHD5 is significantly larger (~ 70 residues) and could regulate protein–protein interactions. Closer examination of the model indicated that S2 interacts with S4 and S5 segments. The identification of structural features in this RMSF model of ABHD5 highlighted the structural features that could be important in mediating its complex functions. For instance, S3 and S4 possess a dynamic and lid-like domain that might participate in reversible interactions with PLIN and PNPLA family members.

### Identifying ABHD5 binding surfaces

#### The main functional-pocket of lipase activation

**Figure 3:**
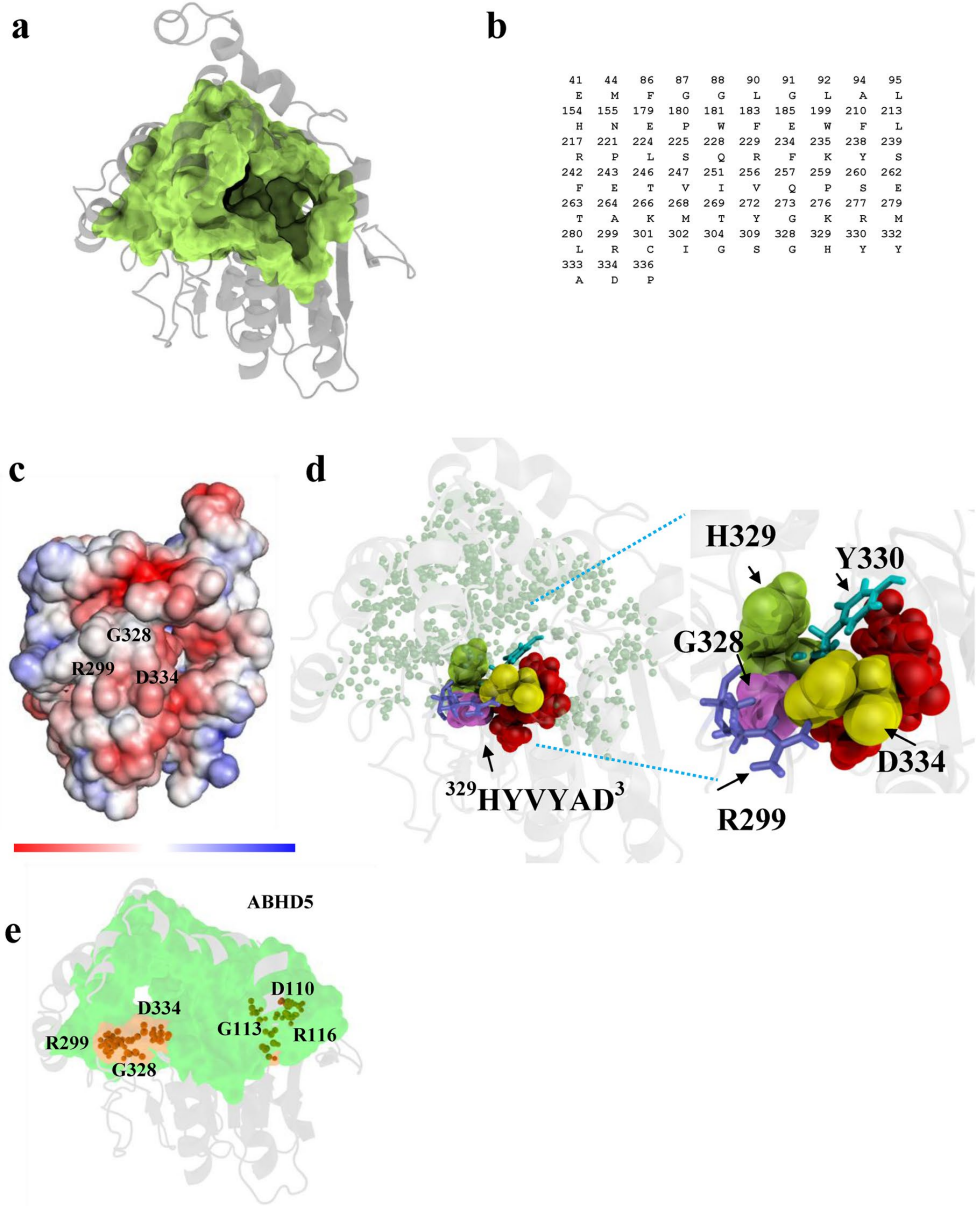
Shape analysis on ABHD5 surface. (**a**) Using the 3D weighted alpha shape with the flow algorithm, shape analysis was performed on the ABHD5 refined structural model. The binding pocket of ABHD5 was identified and assessed for the geometric measurement of molecule volume, solvent accessible area and the number of pocket residues in (**b**). The spatial pattern of the ABHD5 binding pocket represents a novel binding surface in ABHD family. The binding pocket possesses the key residues R299, G328 and D334 that play a crucial role in activating ATGL lipolysis. (**c**) The biochemical and biophysical properties of the binding pocket reflects on the computed electrostatic potential surface. The color key ranges from − 5 kT/e (red) to 5 kT/e (blue). The neighborhood of R299, G328 and D334 exhibits a stronger negatively charged surface that is a hotspot or interface of interacting with positively charged binding partners. (**d**) As the geometric center of R229GD surface patch, D334 is clustered spatially with R299, G328 (pink), H329 (green), Y330 (cyan) and V^331^Y^332^A^333^ (red) buried on the binding pocket (light green dots). The close up view showed the geometric orientation of the surface patch R^299^G^328^D^334^ while positively charged D334 (yellow spheres) is stabilized with negatively charged R299 (blue sticks) with a salt-bridge. (**e**) The functionally important residues (R299, G328 and D334) and (R116, G113 and D110) are identified and characterized on the binding surfaces of ABHD5.

Shape analysis identified the main binding pocket of ABHD5 consisting 63 residues with a solvent accessible area of 1812 Å^2^ and a molecular volume of 2586 Å^3^ (Fig. 3a,b). Among the binding pocket residues, 18 are polar, including four Ser, one Asp, six Glu, three Lys, and four Arg residues (Fig. 3b). We defined pocket α as the pocket that involves in ABHD5 function. Geometrically, the model reveals that the binding pocket has three entrances gated by R217 and R299 for the second and third entrances, respectively, whereas the first entrance gated by R116 (Fig. 2c) is channeled to the second largest binding pocket θ (see definition below). Moreover, the pocket θ contains a large hydrophobic surface patch enriched with Gly residues located near the pocket entrances, which could be involved in binding phospholipids and selectivity on the membrane surface^30^. To interrogate each of the pocket residues, we assessed the physicochemical attributes (e.g., charge and hydrophobicity) of each residue mapped to ABHD5 surface (Fig. 3c). The pocket entrance gated by R299 manifests a negatively charged interface that potentially interacts with the positively charged interfaces of binding partners.

We next explored residues on ABHD5 surfaces to delineate a site-specific mechanism underlying ATGL activation. The negatively charged amino acid residues including Arg, and Lys could be utilized for binding membrane^31^ and positively charged (polar) residues including Asp and Glu might play important roles in salt-bridges and protein stability. With the 63 identified pocket residues of the main binding pocket Fig. 3a,b, we primarily focused on the four Arg pocket residues (R217, R229, R277, and R299) and the only one Asp (D334), because of their physicochemical attributes in protein function, stability and protein–protein interactions.

Using the weighted Alpha Shape in structural analysis, we identified the one Arg (R299) spatially close to the geometric center of Asp (D334) as shown in Fig. 3d. In our prior study, we demonstrated that R299, G328 and D334 forms a functional surface (Fig. 3d) of pocket α on the binding pocket. We characterized the pocket α with the site-specific key residues R299, G328 and D334 clustered (Fig. 3e) in the spatial pattern, and uncovered their functional role of activating ATGL lipolysis^9^. To characterize the biochemical role of D334, we assessed the physiochemical attribute of the carboxyl side-chain of Asp (D) on this surface. To do so, we considered the corresponding spatial position of D334 of homologs across species in the entire structure database. In brief, we conducted a large-scale surface matching of pockets collected in our pocket database^14,24,32–36^ to identify the substitution patterns of a pocket residue D334 that have evolved naturally. We identified the five potential amino-acid substitutions of Asp (D), including (E, G, H, N, and Y) that potentially alter its physicochemical attribute in the binding network.

**Figure 4:**
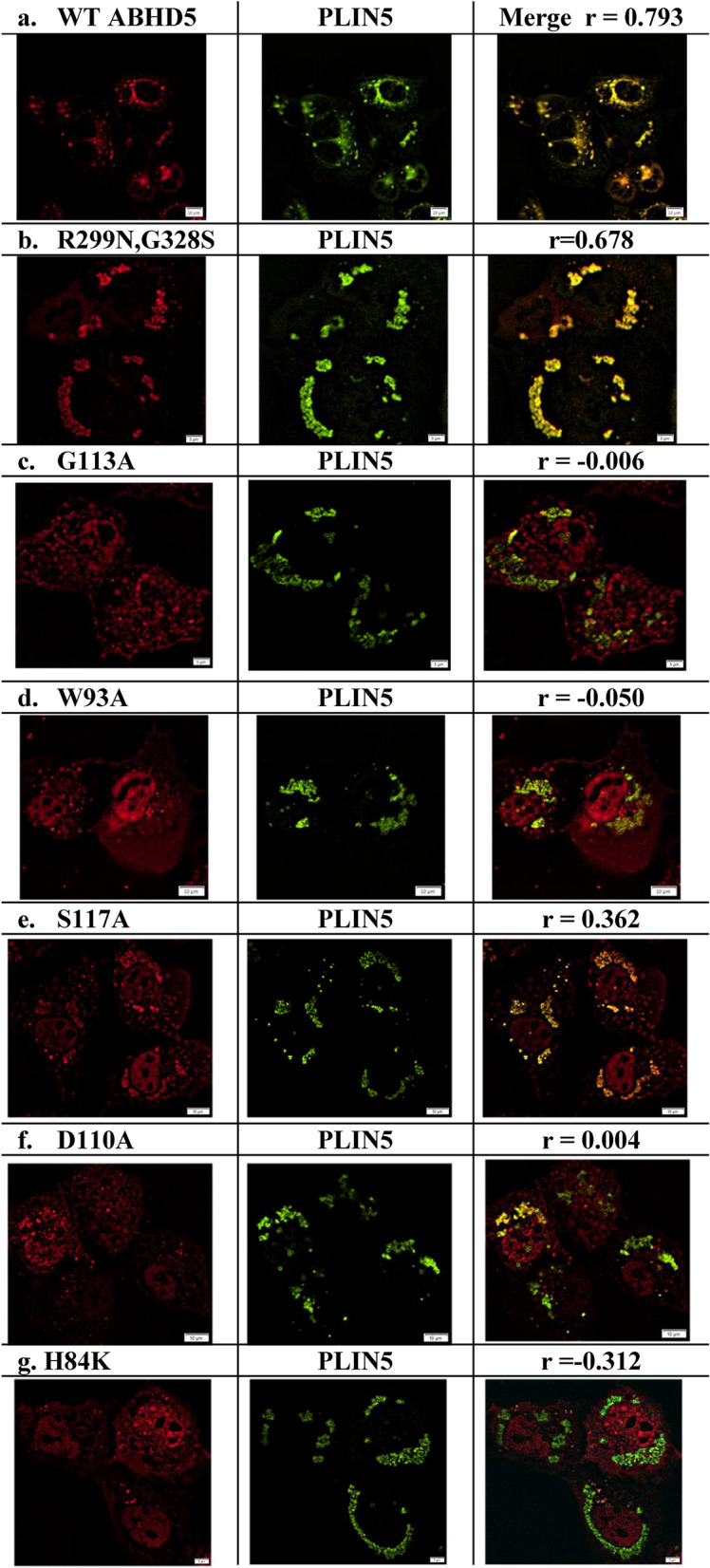
Subcellular localization of ABHD5 mutants in lipid droplet formation assays. Cos7 cells were cotransfected with cherry-tagged ABHD5 or ABHD5 mutants, PLIN5-EYFP and ATGL-ECFP and exposed to oleic acid overnight. Cells were fixed and imaged by confocal microscopy using a ×60 water immersion objective. Representative regions of interest containing triply-transected cells are shown. All mutants were defective in preventing lipid droplet formation as indicated by clusters of PLIN5-containing LD. Colocalization between ABHD5 and PLIN5 was performed using Pearson’s regression (r) in the cellSens software package.

Experimentally, we found that D334G and D334N^9^ significantly abrogated ATGL activation without affecting protein expression. Furthermore, we found that D334A mutation also eliminates ATGL activation, but this mutation also reduced protein expression and PLIN binding. In contrast, ABHD5 function remained largely intact in the D334E mutant, which maintains the negative charge. Site-directed mutagenesis of R299 and G328 abolishes PNPLA2/ATGL lipolysis activation by ABHD5, whereas reconstructing this pocket in ABHD4 confers lipase activation to this close paralog^9^. In colocalization analysis, we transfected Cos7 cells with PLIN5-EYFP and PNPLA2/ATGL-ECFP in the presence of oleic acid. In Fig. 4a, we observed the colocalization between WT ABHD5 and PLIN5 as a control. We assessed the colocalization between ABHD5 and PLIN5 using Pearson’s regression (r). Compared to WT ABHD5 (r = 0.793), we consider r < 0.5 as an indication of partly defective colocalization, and a strongly defective case if r < 0.1. We found that the double substitutions of R299N and G328S do not significantly affect LD targeting or PLIN binding (r = 0.678, Fig. 4b). Taken together, the triad of R299, G328, and D334 on this novel surface of ABHD5 has been evolutionarily shaped for activating PNPLA2/ATGL lipolysis, which manifests a unique structural signature of ABHD5 in proteins.

#### Identification of a conserved functional pocket

**Figure 5:**
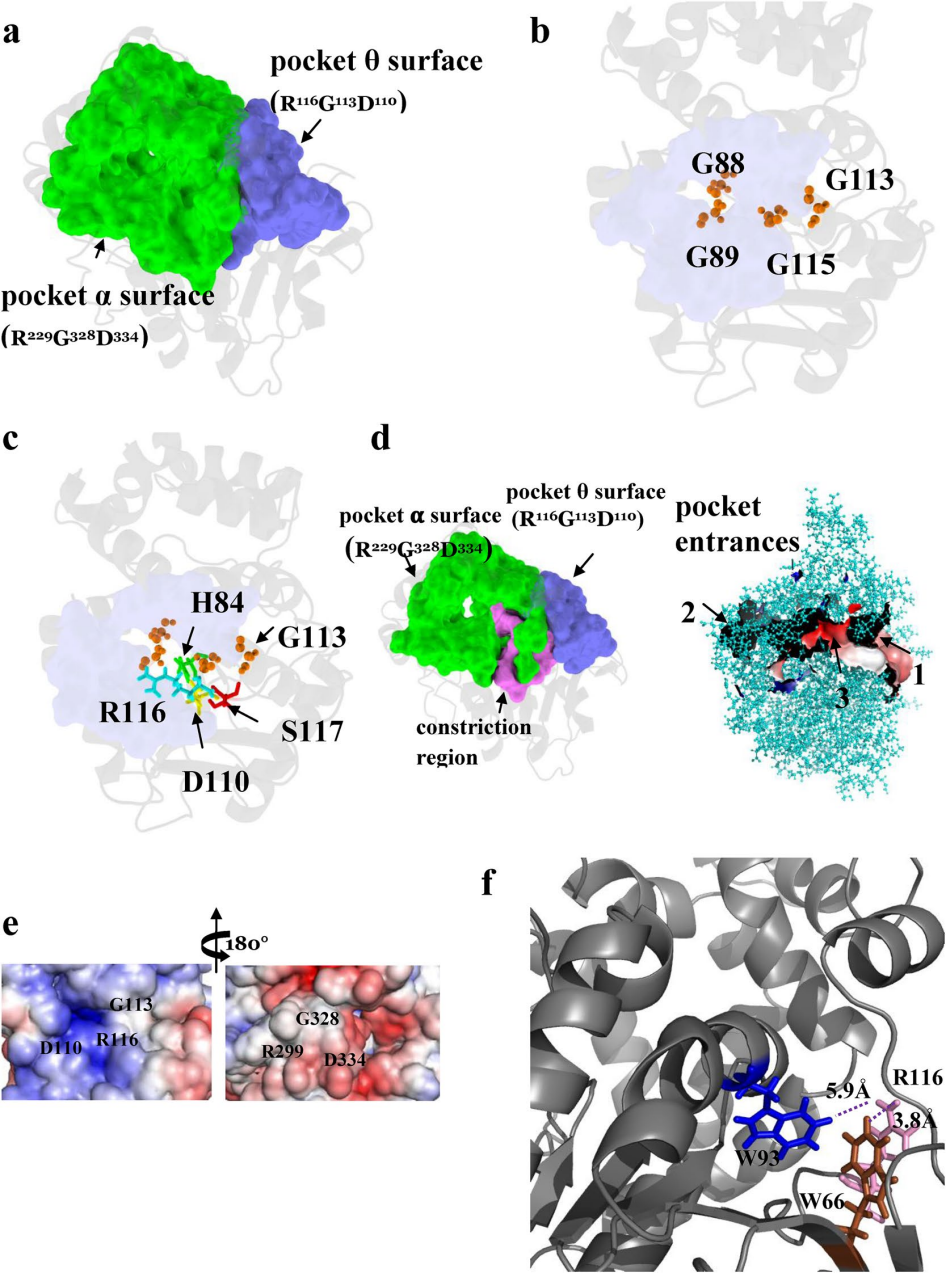
Identification of ABHD5 binding pockets. (**a**) The binding pocket θ (R^116^G^113^D^110^, blue) was identified spatially attached to the main binding pocket α (R^299^G^328^D^334^, green). (**b**) Closer examination of the binding pocket θ (R^116^G^113^D^110^) indicated Gly-rich surface might recognize the phosphate moiety of phospholipid on the LD surface (**c**) Embedded within the binding pocket θ (R^116^G^113^D^110^), the putative key residues S117, G113 and D110 are spatially clustered with R116 and H84. (**d**) The geometric locations of the functionally important residues R299, G328, and D334 are situated closely on the pocket α which synergistically cooperates with the pocket θ that harbors a surface patch of R116, G113, and D110. Connecting the binding pockets (pocket α and θ), the constriction region (pink) could play a role of gating the channel that accommodates the acyl-tail of ligand/substrate entering the main pocket α from its entrance-1 or pocket θ from entrance-2. (**e**) R^116^G^113^D^110^ is a newly identified surface patch in the present study, while R^299^G^328^D^334^ has been identified and experimentally tested to active ATGL lipolysis in our previous study. Electrostatic potential were computed on R^299^G^328^D^334^ and R^116^G^113^D^110^ surface patches, each clustered in the same spatial pattern. R^299^G^328^D^334^ surface patch exhibits negatively charged neighborhood, whereas R^116^G^113^D^110^ displays positively charged surface patch. (**f**) The guanidinium group of positively charged residue R116 interacts with the aromatic rings of W93 and W66 within a range of 5.9 and 3.8 Å, respectively.

ABHD5 is a complex protein that differentially interacts with effector and repressor proteins in a ligand-dependent fashion. Therefore, we hypothesized that ABHD5 contains additional functional surfaces related to protein–protein interactions and ligand binding. Our model revealed a putative binding pocket that is connected to the main functional pocket on the ABHD5 surface. We defined pocket θ as a pocket that might participate in ABHD5 function. This binding pocket is composed of 20 pocket residues with a solvent accessible area of 287 Å^2^ and a molecular volume of 398 Å^3^ (Fig. 5a,b). The binding pocket contains a Gly-rich cluster (G87, G88, G89, G91 G113, G115 and exhibits an amphipathic helix composing of the aromatic rings of H84, F86 and W93 and lipophilic Leu residues including L90, L92, L95, and L100. Of G87, G113 and G115. In colocalization analysis by confocal imaging, we found that alanine or serine mutation of G113 completely disrupted membrane targeting, interactions with PLIN1 and PLIN5 (Fig. 4c), as well as activation of PNPLA2/ATGL-dependent lipolysis. Similarly, W93A mutation completely disrupted both PNPLA2/ATGL activation and LD targeting (Fig. 4d,). These observations indicate that pocket θ which contributes to core functions of ABHD5, including LD targeting, interactions with PLIN proteins, and activation of PNPLA2/ATGL.

Shape analysis revealed that the predicted binding pocket including H84, F86, D110, G113, R116 and S117 (Fig. 5c) matches with a highly conserved domain among lipases (e.g., MGLL in Fig. 2d) and phospholipase families. Interestingly, this binding pocket encompasses a structurally conserved signature of R116, G113 and D110 on pocket θ among ABHD5, ABHD4, and related lipases. As shown in Fig. 3e, we identified the geometric locations of the subgroups of functionally important residues (R299, G328 and D334) and (R116, G113 and D110) on pocket α and θ, respectively. We further characterized the pocket θ with the identified key residues R116, G113 and D110 clustered in the spatial pattern and attempted to explore their putative biological roles on the LD membrane by constructing ABHD5 mutants. Indicated by electrostatic surface potential in Fig. 5e, the pocket entrance of pocket θ gated by R116 displays a stronger positively charged interface that could be important in binding negatively charged phospholipids of LD membrane. In comparison of the electrostatic surface of R^116^G^113^D^110^ to that of R^299^G^328^D^334^ reveals that pocket θ and α manifest distinct physicochemical features. We hypothesize that the positively-charged surface patch (Fig. 5e) of pocket θ recognizes negatively charge phosphate moiety of a phospholipid. Moreover, the guanidinium group of R116 could play multiple roles including building hydrogen bonds with D110 and Ser117 and cation-π interactions with the aromatic ring of W93.

**Figure 6:**
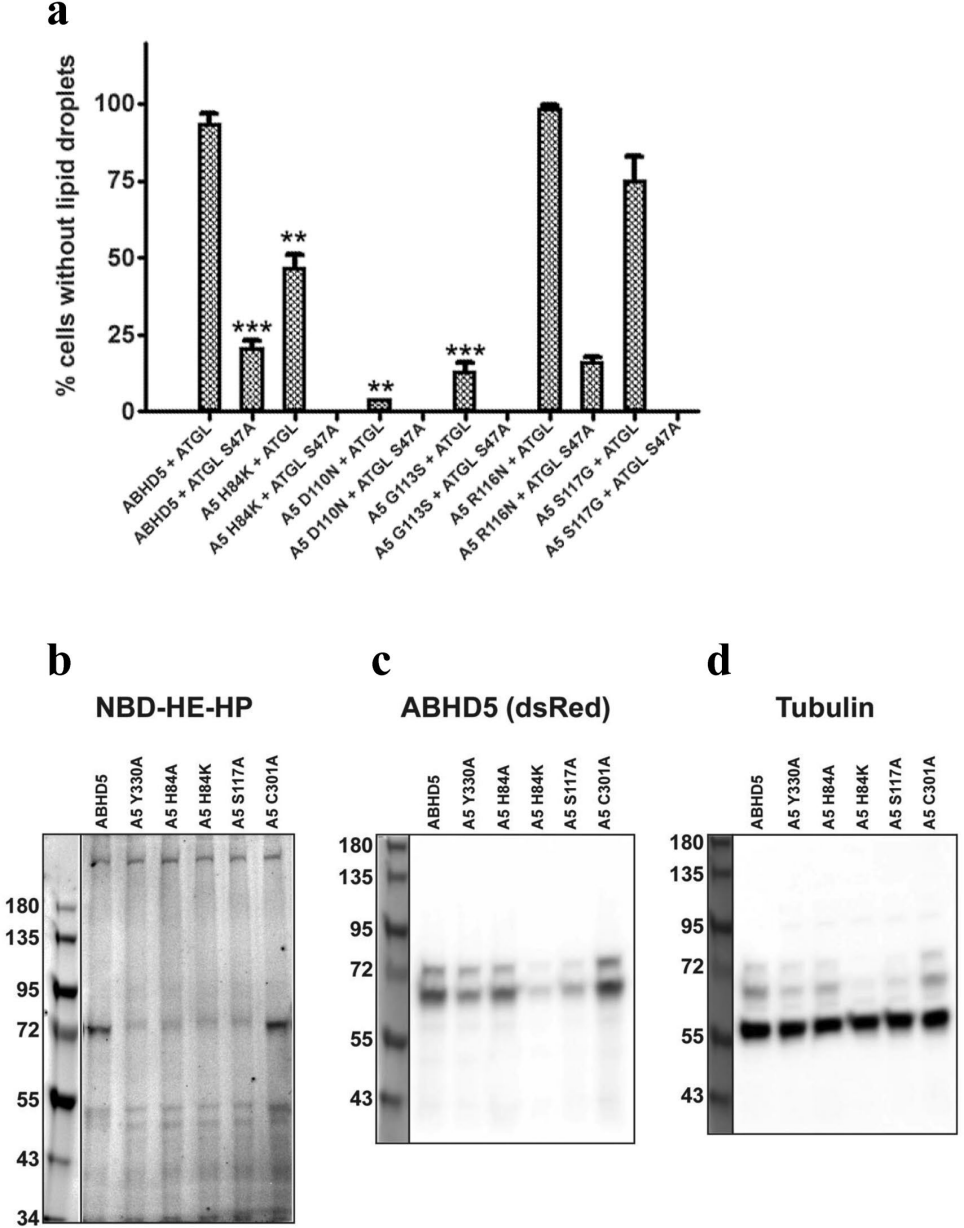
Lipid droplet formation and NBD-HE-HP affinity probe labeling in transfected Cos7 cells. (**a**) Cos7 cells transfected as described in “Methods and data” were lipid loaded and scored for the presence of lipid droplets by blinded analysis. Only cells visibly expressing all three transfected proteins (ABHD5 or ABHD5 mutant-mCherry, Plin1-EYFP, and ATGL or ATGL S47A-ECFP) were scored. Results are mean ± SEM from three independent experiments. **p < 0.01 compared to wild-type ABHD5/ATGL; ***p < 0.001 compared to wild-type ABHD5/ATGL. (**b**) Cos7 cells transfected with cherry-tagged ABHD5 or ABHD5 mutants were labeled with the affinity probe NBD-HE-HP (50 µM) then labeled proteins were resolved by SDS-PAGE and imaged as described in “Methods and data”. The gel was then immediately transferred to a PVDF membrane and cherry-tagged proteins were detected with dsRed antibody (**c**), then immunoblot was reprobed with α/β-tubulin antibody (**d**). One of two independent experiments with similar results is shown.

To assess the functional significance of these amino acids we examined the effects of ABHD5 point mutations on PNPLA2/ATGL activation in transfected Cos7 cells. PNPLA2/ATGL activation was assessed by comparing lipid droplet accumulation in the presence of ABHD5 and WT PNPLA2/ATGL versus ABHD5 and lipase-dead S47A ATGL (negative control). Compared to WT ABHD5, we found that point mutation of binding pocket residues G113S, S117A or D110N suppressed activation of PNPLA2/ATGL (Fig. 6a). These ABHD5 mutations also disrupted colocalization with PLIN5 (Fig. 4c,e,f). In contrast, R116N disrupted LD-targeting, but not lipolysis activation (Fig. 6a) or interactions with PLIN1 or PLIN5. We concluded that ABHD5 manifests a novel surface patch of R^116^G^113^D^110^ with functionally important residues H84, W93 and S117 to engage in LD-targeting and PLIN-binding.

The structural prediction and experimental results suggest that the binding pocket θ cooperates with the main binding pocket α (Fig. 5a). The binding pocket θ is required to carry out LD-targeting with PLIN and to prepare the biophysical settings of the main functional pocket α activating PNPLA2/ATGL. In this regard, our model further suggests that the pocket θ residues H84, D110, G113, R116, S117 might carry out uncharacterized reactivities required for interfacial activation (Fig. 2d).

#### A constriction region in ABHD5 connects the two binding pockets (pocket α and θ)

A unique feature of ABHD5 is a constriction zone that contacts or overlaps pocket residues H84, F86, G87, G88, G89, L90, G91, L92, W93, and L95 (Fig. 5d). This boundary region is mostly constructed by highly conserved Gly and Leu residues, and is gated by R116, W93 and F86. Close examination of the pocket residues and spatial neighborhoods surrounding the constriction region revealed an amphipathic helix of ~ 20 residues (84–105) located in the S2 segment. H84 is a highly conserved residue interacting with a cluster of Gly residues that might mediate phospholipid recognition in a hydrogen bonding network. To characterize the spatial position of H84 that has been substituted in evolution, we utilized shape analysis of surface matching against our database of spatial patterns. We identified four amino acid substitutions including Q, L, P, and Y that might be allowed in the binding network, whereas K and R appear to be disallowed. Consistent with this analysis, mutation of H82K or H82R in humans (H84 in mouse) causes CDS in humans (see Table 1; Fig. 6a). Experimentally, mutation of H84K in mouse ABHD5 completely disrupted LD targeting, PLIN association and ATGL activation (Fig. 4F). In colocalization analysis, the H84K mutant of mouse ABHD5 was excluded from PLIN5-containing LD (Pearson’s regression r = − 0.312). Interestingly, we also found that H84A mutation does not significantly disrupt function, indicating positively charged residues (R or K) at H84 is deleterious to ABHD5 function.

**Table 1:** CDS-causing non-synonymous single nucleotide polymorphisms in ABHD5.

Residue variant	Geometric location in our model	Structural segment in our model	Experimental tests on mouse	Structural interpretations
H82R (Mouse: H84K)	pocket	S2	Severely affects 1. PLIN-binding (Fig. 4g) 2. LD-targeting (Fig. 4g) 3. NBD-labeling (Fig. 6b–d)	H84: a pocket residue (Fig. 8) 1. H84 is involved in PLIN1-binding 2. H84K disrupts NBD-HE-HP binding (Fig. 7d)
S115G (Mouse: S117A)	pocket	S2	Severely affects 1. PLIN-binding (Fig. 4e) 2. LD-targeting (Fig. 4e) 3. NBD-labeling (Fig. 6b–d)	S117: a pocket residue (Fig. 8) 1. S117 is involved in PLIN1-binding 2. S117A disrupts NBD-HE-HP binding (Fig. 7d)
Q130P (Mouse Q132)	surface	S2	NA	Q130: a surface residue (Fig. 8) Q130P likely causes structural instability or folding issues
E260K (Mouse E262K)	pocket	S4	Severely affects^10^ 1. PLIN-binding 2. LD-targeting 3. NBD-labeling	E260: a pocket residue (Fig. 8) 1. E260 is involved in PLIN-binding 2. Mouse E262K charged inversion disrupts PLIN-binding and NBD-HE-HP binding (Fig. 7c) 3. Mouse E262A disrupts NBD-HE-HP binding but not PLIN-binding (Fig. 7c)

In our RMSF model, we found that the S2 segment (composed of the constriction region) interacts with S4 and S5 segments, which regulate pocket entrances shown in Fig. 5d. This structural relationship indicates that this constriction region connects the two binding pockets (α and θ). Geometric calculations revealed that F86 of the S2 segment interacts with the Y272 of S4, and W93 of S2 is oriented closer to stabilize the charged residues R116 and D110, while L92 of S2 simultaneously interacts with the Y332 and F339 of the S5 segment, indicating that the lid-like domain of the S4 segment frequently interacts with the amphipathic helix in S2 to regulate access to the binding pocket θ. These observations suggest that F86, W93 and R116 coordinate interactions with the constriction region so that they could function to gate the entrance of the binding pocket θ channeling into the main binding pocket α, when ABHD5 is bound to LD. In support of this hypothesis, we found that W93A mutation blocks association with PLIN5 on LD (Pearson’s regression r = − 0.050 in Fig. 4C). This single mutation strongly indicates that W93 plays a crucial role not only in targeting LD surface but also might stabilize R116 on the surface patch of pocket θ via cation–π interactions (Fig. 5f).

### Applying affinity probes to interacting with the binding pocket residues of ABHD5 by NBD-HE-HP

**Figure 7:**
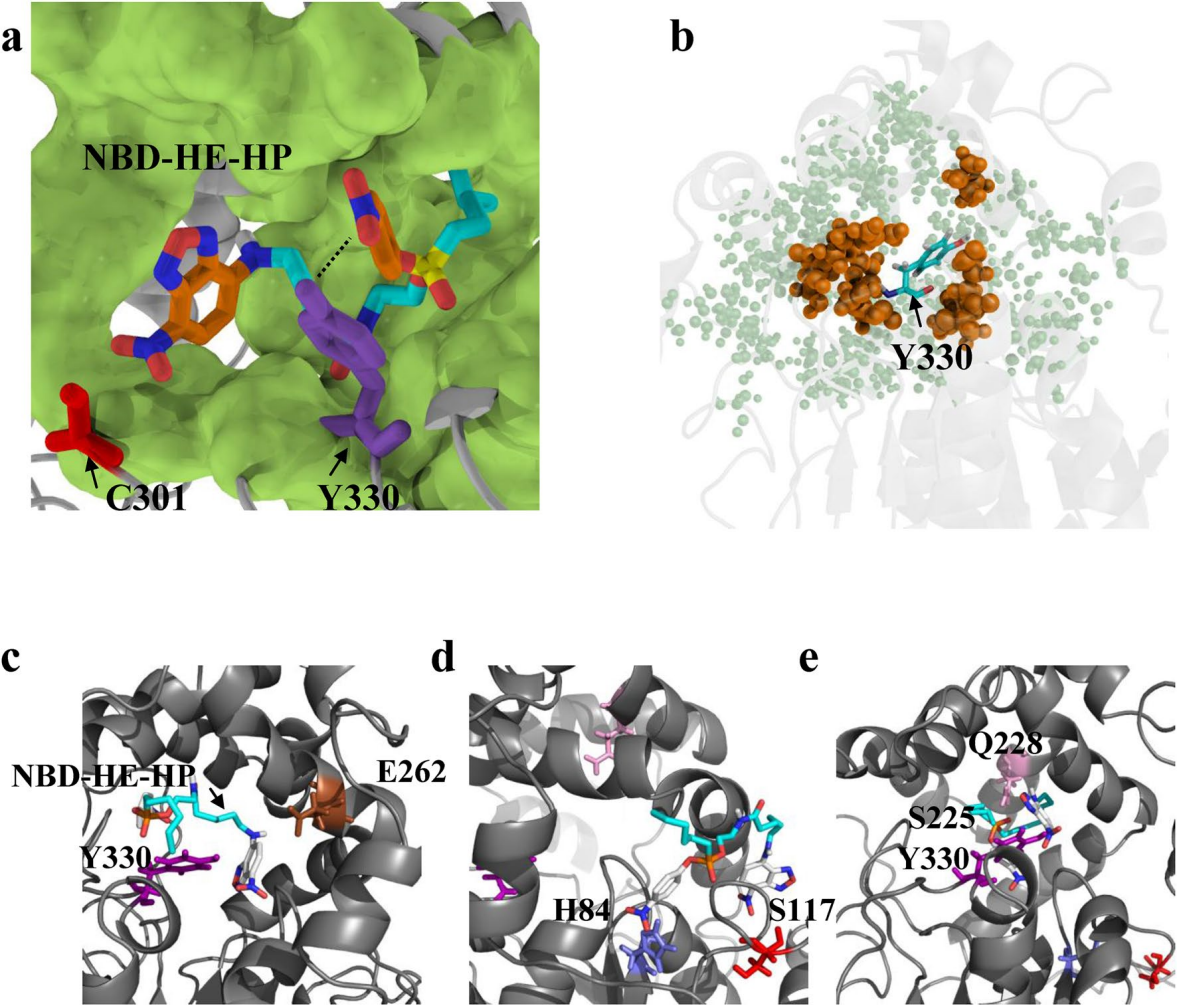
Affinity probe of identifying ABHD5 binding sites. The docking pose underpinned by LC–MS/MS analysis demonstrated the affinity probe forms an adduct with Y330. (**a**) The binding pose of NBD-HE-HP (sticks) was predicted in the binding pocket α (R^299^G^328^D^334^). The nitrophenol moiety of NBD-HE-HP was docked spatially close to the hydroxyl group of Y330 (purple) and the NBD moiety was placed near the neighborhood of C301 and Y330 of H^329^YVYAD^334^ motif (orange spheres) in a close-up view in (**b**), whereas the acyl-tail (cyan sticks) penetrated the tunnel that connects the binding pockets (α and θ). (**c**) The NBD moiety of NBD-HE-HP has a higher affinity of binding to Y330 and E262, S117 and H84 as shown in (**d**), S225 and Q228 in (**e**). Our experimental tests showed that mutations on these identified residues lead to disrupting NBD-HE-HP-labeling.

We recently demonstrated that ABHD5 contains binding sites for acyl-Coenzyme A and synthetic ligands. Furthermore occupancy of the site by acyl-CoA promotes a conformation of ABHD5 that binds PLIN proteins and prevents PNPL2/ATGL activation, whereas occupancy by synthetic ligands SR4995 or SR4559 prevent PLIN interaction and promote ATGL activation. To explore how ligands interact with ABHD5, we took advantage of our discovery that the covalent affinity label NBD-HE-HP bonds covalently to Y330^9,12^ (Figs. 6b–d, 7a). As mentioned above, Y330 is present within the binding pocket α and the pocket is sufficiently large (2586 Å^3^) to accommodate the NBD-HE-HP molecule (Fig. 7a,b).

To better determine the binding surface of ABHD5, we docked NBD-HE-HP into the geometric center of the predicted binding surface in molecular dynamic simulations (see “Methods and data”). As shown in Fig. 7a, one pose placed the reactive nitrophenol moiety of NBD-HE-HP the near Y330 in the H^329^YVYAD^334^ motif (Fig. 7b). As docked onto Y330, NBD-HE-HP arrives in the neighborhood of E262 and C301 within the binding pocket (Fig. 7c). Dynamic modeling also placed the 7-nitro-2-1,3-benzoxadiazol-4-yl substructure (i.e., NBD moiety) near the hydroxyl moiety of a Ser225 with multiple potential poses at (H84, S117, Fig. 7d), or at (Q228, S225, Fig. 7e) with the acyl-tail moiety of NBD-HE-HP oriented into the hydrophobic constriction region that connects the two binding pockets (pocket α and pocket θ). Docking and geometric calculations further predicted that NBD-HE-HP also interacts with the polar residues such as H84 and S117 on the binding pocket θ (Fig. 6b–d). Consistent with the modeling data, Q228A (Fig. 7e) mutation significantly disrupted NBD-HE-HP labeling without affecting LD targeting or PLIN interactions. Mutations of H84 and S117 disrupted LD targeting and abolished labeling by NBD-HE-HP. In our previous study, our experimental data uncovered that E262K mutant severely abrogates PLIN interactions and NBD-HE-HP labeling, whereas E262A only affects NBD-HE-HP labeling. Collectively, our findings indicate that pocket α (Q228, Y330 and E262) and pocket θ (H84 and S117) residues are cooperatively involved in the binding sites for protein binding partners and potential endogenous ligands.

### Identifying the structural locations of CDS-causing mutations in ABHD5

**Figure 8:**
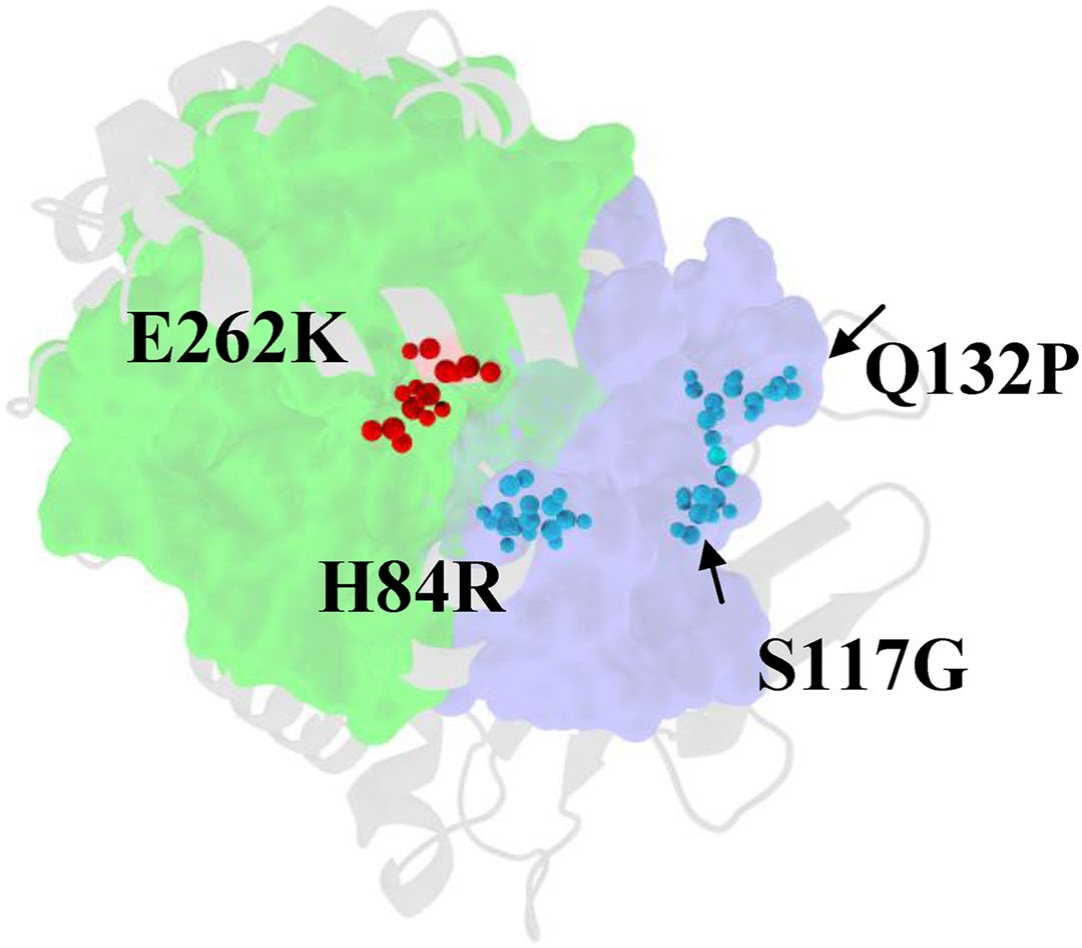
Structural identification and experimental characterization of human CDS-causing mutations on ABHD5. Human ABHD5 harbors CDS-causing mutations H82R, S115G, Q130P, E260K, G271R (corresponding to mouse H84, S117, Q132, E262 and G273) that were structurally located on the binding pockets (pocket α and θ) or surface, whereas R234N and R280N (mouse R236 and R282, see Table 1) were reported pathogenic nsSNPs located on ABHD5 surface.

Our refined model also provides new structural insights into disease-causing mutations of human ABHD5, including H82R^37^, S115G^38^, Q130P^39^ and E260K^39^ (corresponding to mouse H84, S117, Q132, E262 in mouse ABHD5) that result in CDS (OMIM:275630, Online Mendelian Inheritance in Man) (see Table 1). Our model also classifies disease-causing mutations in ABHD5 based on their geometric locations, e.g., surface, pocket, and core^14,40,41^. Shape analysis revealed that the majority (> 97%) of human disease-causing non-synonymous single nucleotide polymorphisms (nsSNPs) in protein coding regions located in pockets and protein–protein interfaces^14,40,41^. As mapped onto our 3D model in (Fig. 8), the structural locations of the CDS-associated nsSNPs were geometrically classified. For instance, shape analysis revealed that H84, and S117 are located on the pocket θ, E262 on the pocket α, and Q132 on the shallow surface of S2 segment.

We sought to identify human nsSNPs that might exhibit a spectrum of susceptibility to CDS caused by pathologically defective ABHD5. To assess the mutational influence of a mutant, we identified its structural location and characterized its physiochemical attributes that might influence ABHD5 functions. Importantly, experimental tests on the mouse ABHD5 (Table 1) were performed to evaluate mutational effects elicited by selected nsSNP(s). Our data showed that the pocket residue E262 interacts with NBD-HE-HP (Fig. 7c), indicating a potential binding site for endogenous ligands or/and for PLIN or ATGL via electrostatics. Our experimental data^10^ support that the charge inversion of E262K mutant might alter this crucial binding interface of binding partners, thereby interrupting interactions with ATGL and PLIN proteins. In contrast, E262A mutant prevented NBD-HE-HP labeling without affecting interactions with ATGL or PLIN. The charge inversion of E262K is projected on the surface and consequently perturbs the binding with PLIN. In contrast to E262A, the substitution that introduced the charged residue in place of neutral or short side-chain ones does not drastically and constitutively perturb the binding interface. Moreover, E262 has functional significances on the main binding pocket α. The mutation of E262A suppressed the binding with ligands such as NBD-HE-HP mostly because the short side-chain of A262 does not confer favorable atomic contacts with NBD-HE-HP (Fig. 7c).

It appears that substitution of the imidazole ring of H82 by positively charged side-chain or the polar side-chain of S115 by a short hydrophobic side-chain residues increase susceptibility to CDS. As a pocket residue, S117 of mouse ABHD5 was oriented on the surface of the pocket θ (Fig. 5c). Our experimental data (Figs. 4d, 6a) showed that S117A substitution impaired the mouse PNPLA2/ATGL lipolysis, since the removal of the side-chain of S117 noticeably inhibited LD-targeting and the binding to PLIN. In addition to the impaired LD translocation, the mouse S117A mutant abolished NBD-HE-HP labeling (Fig. 6b–d, 7d). Moreover, the human CDS mutations (Table 1) drastically alter physiochemical properties of these pocket residues. For example, the imidazole moiety of a pocket θ residue H82 of human ABHD5 mutated to the guanidinium group with positive charge of R82 might reduce the efficiency of the charge relay system between H82 and D108 or alter electrostatics to disrupt the interactions with PLIN. We found that the pathological CDS is highly associated with the mutational defects on targeting PLIN5-containing LD and NBD-HE-HP labeling detected in our experimental tests. Similarly, the substitution of either K or A at H84 of mouse ABHD5 blocked NBD-HE-HP labeling (Fig. 6b–d). In support of structural analysis, our experimental data (Figs. 4g, 6b–d) unequivocally showed that the nsSNPs of H84 and S117 oriented and juxtaposed within the pocket θ (Figs. 5c, 8d) that account for disease-causing defects in ABHD5 structure.

## Discussion

ABHD5 arose in vertebrates following gene duplication, loss of serine hydrolase activity, and evolution of novel surfaces that mediate protein–protein interactions and allosteric regulation by endogenous metabolic ligands^12^. Importantly, ABHD5 has been implicated in diverse biological functions, including triglyceride mobilization and sequestration, skin barrier formation and tumor suppression, suggesting involvement of metabolic control beyond lipolysis regulation. Indeed, growing evidence indicates that ABHD5 might be best characterized as a complex metabolic transducer that integrates extracellular and intracellular signals to regulate the trafficking and activity of PNPLA family members. Thus, it is well-known that ABHD5 regulates the activity of PNPLA2/ATGL on lipid droplets in adipocytes via phosphorylation-dependent interactions with PLIN1^42^. Furthermore, ABHD5 contains binding pocket for long chain acyl-CoA and synthetic ligands that control its interaction with PNPLA and PLIN family members^8,12^.

To facilitate understanding of the functional and structural mechanisms of these biological activities, we explored the binding surfaces that confer this complex regulation. We computed the dynamic shapes of ABHD5 in the simulations of multiple time scales. Our structural analysis identified major binding surface that could be divided into two connected binding pockets (pocket α and pocket θ in Fig. 5a) which cycle among transient states (Fig. 1f). The experimental data strongly indicate that each of the identified binding pockets carries out its biological function, and the two binding pockets are functionally interconnected and cooperate with each other to allosterically regulate PNPLA2/ATGL activity. That is, the pocket θ first involved in LD-targeting and membrane binding, whereas the pocket α subsequently activates PNPLA2/ATGL lipolysis. Interestingly, their pocket resides might be allosterically regulated by our synthetic compounds and endogenous ligands.

To gain structural insights into how ABHD5 activates PNPLA2/ATGL, we first focused on the binding surface of pocket α at R299, G328, and D334^9^. The binding surface of pocket α exhibits a structural signature for PNPLA2/ATGL lipolysis activation that is not shared by any other known ABHD members. Site-directed mutagenesis of R299, G328 and D334 verified the importance of these residues in PNPLA2/ATGL activation and support the structural relationships of these residues based on shape analysis. Perhaps more importantly, substituting the N290R and S319G in ABHD4, which does not activate PNPLA2/ATGL, was sufficient to confer lipase activation in ABHD4. This discovery of the novel binding surface of pocket α provides a platform for the assessment of gain or loss of function in ABHD5, and further highlights the underlying mechanism of PNPLA2/ATGL activation that might be the fundamental strategy of cells to access the TG molecules within LD.

In addition to the novel binding surface of pocket α for ATGL activation, the present study discovered the surface patch of R116, G113 and D110 of pocket θ that is highly conserved across species. In the Pfam database^43^, the surface composed of the position 80–180 of ABHD5 is highly conserved among the ABHD family members. We also speculate that R^116^G^113^D^110^ on pocket θ could be involved in interfacial activation. In support of this speculation, we showed that mutation of W93 (Fig. 4d) disrupted targeting to PLIN5 on lipid droplets. In addition to W93, our model and experimental data also suggested that the pocket θ harbors functionally important residues including H84, D110, G113, R116 and S117. Consistent with the predictions of the model, H84K, W93A, D110A, and G113S disrupted the ability of ABHD5 to bind lipid droplets, interact with PLIN, and activate ATGL, whereas H84A and S117A markedly inhibits covalent labeling by the affinity probe NBD-HE-HP. In support of the computational identifications, site-specific mutagenesis and cellular imaging were performed on the functionally important residues on the newly discovered R^116^G^113^D^110^ surface patch. The findings led us to layout the configuration on the surface patch of pocket θ that is divided by two sets of functionally important residues to carry out membrane binding. Clearly, the surface of pocket θ is critical for facilitating in the core functions of ABHD5, and likely other closely-related paralogs.

On the ABHD5 surface, we identified R^299^, G^328^ and D^334^ on pocket α for PNPLA2/ATGL lipolysis activation and further discovered R^116^, G^113^ and D^110^ on pocket θ for LD-targeting and PLIN-binding. We concluded that a protein could utilize the triad of R (Arg), G (Gly) and D (Asp) to construct a binding interface to interact with other bioactive molecules. For example, G^113^ might directly influence membrane selectivity and binding on surface while negatively charged D^110^ might contribute to structure stability and protein–protein interaction. Recently, we identified a surface patch of R^357^, G^354^ and D^355^ on human ACE2 (Angiotensin Converting Enzyme 2, a cell membrane receptor) form a critical interface bound and attacked by the Spike proteins of SARS-CoV-2 (Severe Acute Respiratory Syndrome coronavirus) with strong binding affinity^44^. Our experimental data of Spike-binding, cell-based attachment, and in vivo SARS-CoV-2 pseudovirus assays successfully demonstrated that the D355N of human nsSNP and the G354D/Q of primate nsSNP abrogated the Spike-protein binding of SARS-CoV2, indicating a low susceptibility to SARS-CoV2 infections. The structural identification and characterization of the binding interface constructed by Arg, Gly and Asp for protein–protein interactions are qualitatively in agreement with our experimental evaluations.

As mentioned above, our refined model indicates that the pocket α and pocket θ are connected by a constriction region composed of hydrophobic residues. As shown in Fig. 5d, the surrounding boundary surface connects two binding pockets (pocket α and θ) and share the common residues lining the pockets, indicating that they cooperate to carry out multiple ABHD5 functions. For example, the constriction region could regulate access of ligands to the binding pockets. Our geometric calculations in the RMSF model (Fig. 2c) showed that the lid-like domain (from the position 180 to 280 covered by S3 and S4) of ABHD5 interacts the amphipathic helix of S2 that constructs the constriction region. Moreover, the residue contacts between the flexible lid-like domain and the boundary zone gives the clues as to how structure of the lid-like domain might control access to the two main pockets.

Our dynamic model also predicted binding sites for endogenous and synthetic ABHD5 ligands. We previously reported that ABHD5 can be covalently bound by the promiscuous lipase affinity ligand NBD-HE-HP. This labeling can be blocked by endogenous oleoyl-CoA and the synthetic ligand SR4995^12^, indicating the existence of overlapping or interacting binding sites. Proteomic analysis demonstrated that the reactive nitrophenol of NBD-HE-HP forms an adduct with Y330 of ABHD5, which our computational model places within the putative ligand binding pocket α. Furthermore, docking poses in simulations placed the NBD-HE-HP moiety close to Y330 and C301 and mutation of Y330 to Ala greatly disrupted binding of NBD-HE-HP to ABHD5 (Fig. 7a). The active pose further provided structural identification of residues e.g., E262 that might be involved in binding NBD-HE-HP, which implicated intradomain interactions. Our experimental data discriminated the mutational effects between E262K and E262A, and further identified the main binding attribute via electrostatics with binding partners such as PLIN, ATGL and binding ligands. Although E262A mutant appears normal in the tests of PLIN binding, it significantly affected NBD-HE-HP labeling. Interestingly, the experimental observations were reproduced in the mutants H84K and H84A which also expressed distinguishable mutational effects. That is, H84K severely abolished both PLIN1 and NBD-HE-HP binding, whereas H84A mutant blocked NBD-HE-HP labeling only. In this regard, it appears that protein–protein interaction has a higher sensitivity to electrostatic binding than protein–ligand interaction.

With these severe mutational effects observed in E262, H84, and S117 mutants, we have been able to identify and characterize the complex interactions of ABHD5 with its partner proteins (PLIN, PNPLA2/ATGL) and ligands. In support of our conclusions, our experimental data and findings indicate that mutations in pocket α and pocket θ confer susceptibility  to human CDS and further suggest critical binding sites for protein interactions and ligand binding. These important findings give structural and functional insights into a site-specific mechanism underlying PNPLA2/ATGL activation and facilitate the understanding of ABHD5 complex molecular functions.

In conclusion, our combination of computational analysis and experimental tests provides a theoretical and functional basis to elucidate ABHD5 molecular functions of fat mobilizing activity. Based on topological shape analysis, we identified two functional pockets on the ABHD5 surface using structural modeling, and geometric computations. We further identified and characterized functionally important residues that are directly associated to ABHD5 molecular functions. We constructed a variety of site-directed mutants to determine their roles in biological activities including LD-targeting, protein–protein interaction, ligand binding and lipolysis activation, using an array of biochemical assays and cellular imaging in live cells. Importantly, we structurally classified ABHD5 nsSNPs based on their geometric locations and characterized their functionally defective roles that are highly associated with human Chanarin-Dorfman Syndrome. From these new findings, our structural model and experimental validation provide biological insights to facilitate the understanding of ABHD5 molecular functions of a metabolic transducer.

## Methods and data

### A computational structural model of ABHD5.

To assess a structure–function relationship of ABHD5, we built a theoretical model for mouse ABHD5 because it has no crystal structure. The initial model is computed based on a soluble epoxide hydrolase (sEH ABHD7, pdb1qo7) of *Aspergillus niger*^45^ as a homology template by Modeller^23,26^. Their sequence identity between ABHD5 and sEH can be as low as < 15%, of sequence identity. Despite their low pair-wise sequence identities, the structural fold (Alpha Beta 3-Layer aba Sandwich, CATH: 3.40.50.1820)^25^ of ABHD5 belongs to the ABHD family and its overall scaffold appears structurally conserved across species, according to CATH fold classification. The model was constructed and evaluated wtih z-score of Discrete Optimized Protein Energy − 0.82 < 0 and a model score of 0.98 > a cutoff of 0.70^26^. Structurally similar to most of family members, ABHD5 is composed of a core of alpha helices and beta sheets with multiple loops connecting them. With this initial model, we performed structural refinement by MD simulations^13,46^ and conducted geometric calculations. ABHD5 primary sequence is the only input, and we sought to probe ABHD5 structural models and identify functionally important residues with our developed structure-based shape analysis.

### Sampling ABHD5 conformations by MD simulations

We utilized a technique of molecular dynamics (MD) simulations and topology-based geometric computations to explore a variety of ABHD5 conformations. Using GROMACS^46^ with AMBER all-atom force field^47^, we computed conformers at a given time in a trajectory. The time evolution of ABHD5 structural conformers contain a diverse of conformations with perturbed local structures. We collected the perturbed conformations at every 100 pico second during the simulations of 30 nano second. The 300 conformers were compared with the initial model to obtain a time series of RMSDs. We obtained putative structural conformations for further structural comparison and shape analysis.

To obtain the major categories of ABHD5 structural models, we performed structural classifications on these models to determine their conformational changes. Using the customized cutoffs with incremental decreases from 3.5 to 1.5 Å of RMSD, we conducted pairwise structural comparisons of conformers. Iteratively, these diverse conformers can be classified into subclasses. Subsequently, we computed six clustered subclasses in a trajectory, after the structural comparisons of conformations at different time steps. Based on their RMSD values of comparison, we further selected a representative close to the average value of RMSD for each classified subclass based on their overall structural conformers. For each ABHD5 conformer, we further conducted shape analysis for the identification of pocket, surface and interior.

### Performing shape analysis on ABHD5 conformations

We conducted shape analysis on these structural conformers in the trajectory based on the Alpha Shape Theory^17–19^. Using the weighted Delaunay triangulation, we triangulated the atomic coordinates of each ABHD5 conformer. We then used the flow algorithm to analytically compute the molecular volumes (MV) and solvent accessible areas (SAA) of putative binding pockets on the ABHD5 surface. In these structural conformers, we computed more than 30 putative pockets on the ABHD5 surface. For each conformer, we further extracted binding pocket residues from each pocket to determine its spatial pattern by CASTp (Computed Atlas of Surface Topography of proteins)^15,16^. We further identified binding pockets by using SplitPocket algorithm (http://pocket.med.wayne.edu/)^14,24^ and characterized their local surface features such as pocket, surface and interior by the Volbl package^14–16,18,19^. As shown in Fig. 1c–e. these structural models in a trajectory contain a mean of 61 binding residues in ABHD5, a mean of molecular volume of 2360 Å^3^. and its corresponding solvent accessible area of 1514 Å^2^. As shown, the MVs in ABHD5 binding pockets have been fluctuated over time. The dynamic shapes of ABHD5 pockets implicates that MD simulations potentially perturb and rearrange the geometric locations of atoms across these structural conformers. We recapitulated conformers and identified their dynamic pockets for each subclass shown in Fig. 1f. We further characterized each residue on a geometric location by evaluating its likelihood of appearing a pocket residue in a trajectory over time (Fig. 1g). Define the likelihood (φ) of a dynamic pocket residue as $\varphi =\frac{\sum_{1}^{\mathrm{n}}p}{n}$, where $p=1$ if a residue is located on a pocket, and $p=0$ if not a pocket residue, which is determined by the geometric location of a residue. The likelihood of a dynamic pocket residue is computed by analyzing the perturbed conformations at every 100 pico during the simulations of 30 nano second ($n=300$).

### RMSF model of ABHD5

To further dissect the predicted binding pocket, we attributed the movements in dynamics to a pocket residue in MD simulations. We performed structural comparisons of conformations at different time steps in the trajectory. Using GROMACS package, we measured RMSF (root mean square fluctuation) of a residue in the simulations. In addition, we collected the pocket residues on the ABHD5 functional surface for RMSF comparisons. Based on the RMSF calculation with a cutoff of 3 Å for separation, we divided a model into five segments with color labels such as red (1–70), yellow (71–150), green (151–220), cyan (221–275) and blue (276–350), which also separated the binding pocket correspondingly.

### Characterizing ABHD5 surface and docking the ligandable pockets with NBD-HE-HP

To assess physiochemical attributes of ABHD5 surface, we conducted the computations of electrostatics potential surface. Using APBS (Adaptive Poisson–Boltzmann Solver)^48^, we focused on the structural features of pocket mouth residues including R116, R217, and R299 to characterize ABHD5 binding surfaces based on electrostatics calculations.

Using affinity probes such as NBD-HE-HP, we explored the ligandable pockets after the identification of binding pockets. We performed docking NBD-HE-HP into the geometric center of each identified binding surface in molecular dynamic simulations, using the AMBER force field^47^. We prepared the topology including partial charges of NBD-HE-HP with the QM/MM utility sqm in antechamber^49^ for molecular docking and simulations. We presented ABHD5 surface and binding pockets with PyMOL (https://github.com/schrodinger/pymol-open-source) and Chimera^50^.

### NBD-HE-HP affinity probe labeling

NBD-HE-HP affinity label was synthesized and purified as described^51,52^. NBD-HE-HP affinity labeling of Cos7 cells (American Type Culture Collection) was performed as previously described^12^. Briefly, cells were transfected with PLIN1 and wild type or mutant ABHD5 then lipid loaded overnight with 200 µM oleic acid. One day after transfection, cells were labeled with 50 µM NBD-HE-HP in serum-free DMEM for two hours at 37 °C. Cells were rinsed with PBS and protein-matched aliquots of cell lysates were separated by SDS-PAGE. After visualization for NBD-HE-HP labeling, unfixed gel was transferred and detected with dsRed antibody (Takara Bio), then reprobed for α/β-tubulin (Cell Signaling Technology). Gels and immunoblots were visualized on an Azure Biosystems C600 Imager (Cyan2 channel for fluorescent visualization of NBD-HE-HP labelled ABHD5).

### Lipid droplet scoring

Lipid droplet scoring was performed as previously described^9^. Briefly, Cos7 cells plated on coverslips in 12-well dishes were transfected with 0.5 μg each/well of mCherry-tagged mouse ABHD5 protein, mouse PLIN1-EYFP, and either mouse ATGL-ECFP or ATGL S47A-ECFP (lipase inactive) using Lipofectamine^®^ and Plus reagent^®^ (Invitrogen) as described by the manufacturer. Cells were then lipid loaded for 16–20 h with 200 μM oleic acid, then fixed with 4% paraformaldehyde. Lipid droplet scoring was performed by an investigator blinded to transfection conditions. For each transfection condition in each experiment 25 or more cells visibly expressing all three proteins were scored. Confocal microscopy was performed using an automated Olympus IX81 microscope equipped with a spinning disc confocal unit, a 60X 1.2 NA water immersion object and a Hamamatsu ORCA Flash CMOS camera. Post-capture analysis was performed in using Olympus cellSens Dimension software. Mutant ABHD5 proteins were made using standard molecular biological methods, and all PCR-generated constructs were confirmed by sequencing.
